# Mitochondrial metabolites predict adverse cardiovascular events in individuals with diabetes

**DOI:** 10.1172/jci.insight.168563

**Published:** 2023-09-08

**Authors:** Jessica A. Regan, Robert J. Mentz, Maggie Nguyen, Jennifer B. Green, Lauren K. Truby, Olga Ilkayeva, Christopher B. Newgard, John B. Buse, Harald Sourij, C. David Sjöström, Naveed Sattar, Robert W. McGarrah, Yinggan Zheng, Darren K. McGuire, Eberhard Standl, Paul Armstrong, Eric D. Peterson, Adrian F. Hernandez, Rury R. Holman, Svati H. Shah

**Affiliations:** 1 Duke Molecular Physiology Institute, Durham, North Carolina, USA.; 2 Duke University Department of Medicine, Durham, North Carolina, USA.; 3 Duke Clinical Research Institute, Durham, North Carolina, USA.; 4 University of North Carolina at Chapel Hill, School of Medicine, Chapel Hill, North Carolina, USA.; 5 Department of Internal Medicine, Division of Endocrinology and Diabetology, Medical University of Graz, Graz, Austria.; 6 Late-stage Development, Cardiovascular, Renal and Metabolism, Biopharmaceuticals R&D, AstraZeneca, Gothenburg, Sweden.; 7 Institute of Cardiovascular & Medical Sciences, University of Glasgow, Glasgow, United Kingdom.; 8 Canadian VIGOUR Centre, University of Alberta, Edmonton, Alberta, Canada.; 9 University of Texas Southwestern Medical Center and Parkland Health and Hospital System, Dallas, Texas, USA.; 10 Diabetes Research Group at Munich Helmholtz Center, Munich, Germany.; 11 Diabetes Trials Unit, Radcliffe Department of Medicine, University of Oxford, Oxford, United Kingdom.

**Keywords:** Cardiology, Metabolism, Cardiovascular disease, Diabetes, Mitochondria

## Abstract

Metabolic mechanisms underlying the heterogeneity of major adverse cardiovascular (CV) event (MACE) risk in individuals with type 2 diabetes mellitus (T2D) remain unclear. We hypothesized that circulating metabolites reflecting mitochondrial dysfunction predict incident MACE in T2D. Targeted mass-spectrometry profiling of 60 metabolites was performed on baseline plasma samples from the Trial Evaluating Cardiovascular Outcomes with Sitagliptin (TECOS; discovery cohort) and Exenatide Study of Cardiovascular Event Lowering (EXSCEL; validation cohort) biomarker substudy cohorts. A principal components analysis metabolite factor comprising medium-chain acylcarnitines (MCACs) was associated with MACE in TECOS and validated in EXSCEL, with higher levels associated with higher MACE risk. Meta-analysis showed that long-chain acylcarnitines (LCACs) and dicarboxylacylcarnitines were also associated with MACE. Metabolites remained associated with MACE in multivariate models and favorably changed with exenatide therapy. A third cohort (Cardiac Catheterization Genetics [CATHGEN]) with T2D was assessed to determine whether these metabolites improved discriminative capability of multivariate models for MACE. Nine metabolites (MCACs and LCACs and 1 dicarboxylacylcarnitine) were associated with time to MACE in the CATHGEN cohort. Addition of these metabolites to clinical models minimally improved the discriminative capability for MACE but did significantly down reclassify risk. Thus, metabolites reporting on dysregulated mitochondrial fatty acid oxidation are present in higher levels in individuals with T2D who experience subsequent MACE. These biomarkers may improve CV risk prediction models, be therapy responsive, and highlight emerging risk mechanisms.

## Graphical abstract

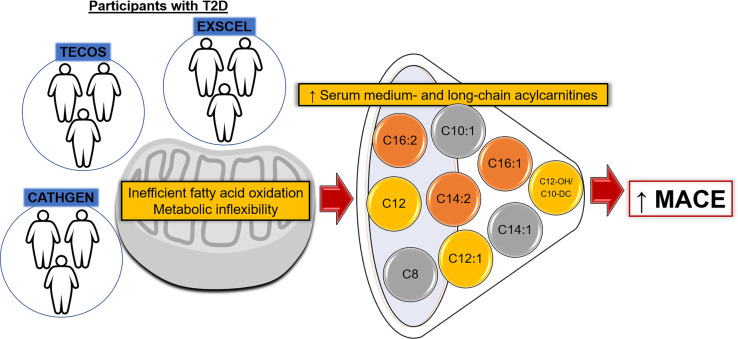

## Introduction

Cardiovascular disease (CVD) remains a primary cause of morbidity and mortality despite advancements in earlier diagnosis and treatment. Individuals with type 2 diabetes (T2D) are at excess risk of cardiovascular (CV) morbidity and mortality compared with those without T2D (1–3); however, there is heterogeneity of risk that is only partially explained by clinical factors.

Cardiac metabolic machinery relies on adequate energy-substrate delivery and intrinsic cardiac metabolism. The contribution of fatty acids and carbohydrates to energy expenditure, termed substrate utilization, is central to metabolic disease, and both CVD and T2D demonstrate perturbations in metabolic homeostasis at organ-specific and systemic levels (4, 5). Emerging technologies have enabled evaluation of this metabolic machinery and have shown utility in the discovery of biomarkers associated with CV risk. Metabolomic profiling has identified that elevated circulating levels of branched-chain amino acids and altered arginine metabolism are associated with coronary artery disease (CAD) (6), and dicarboxylacylcarnitines also appear predictive of major adverse CV events (MACE) (6–8). Levels of branched-chain amino acids and long-chain acylcarnitines (LCACs) are elevated in the setting of insulin resistance and obesity (9–14); however, metabolic pathways linking T2D to MACE remained inadequately understood.

**Figure 1:**
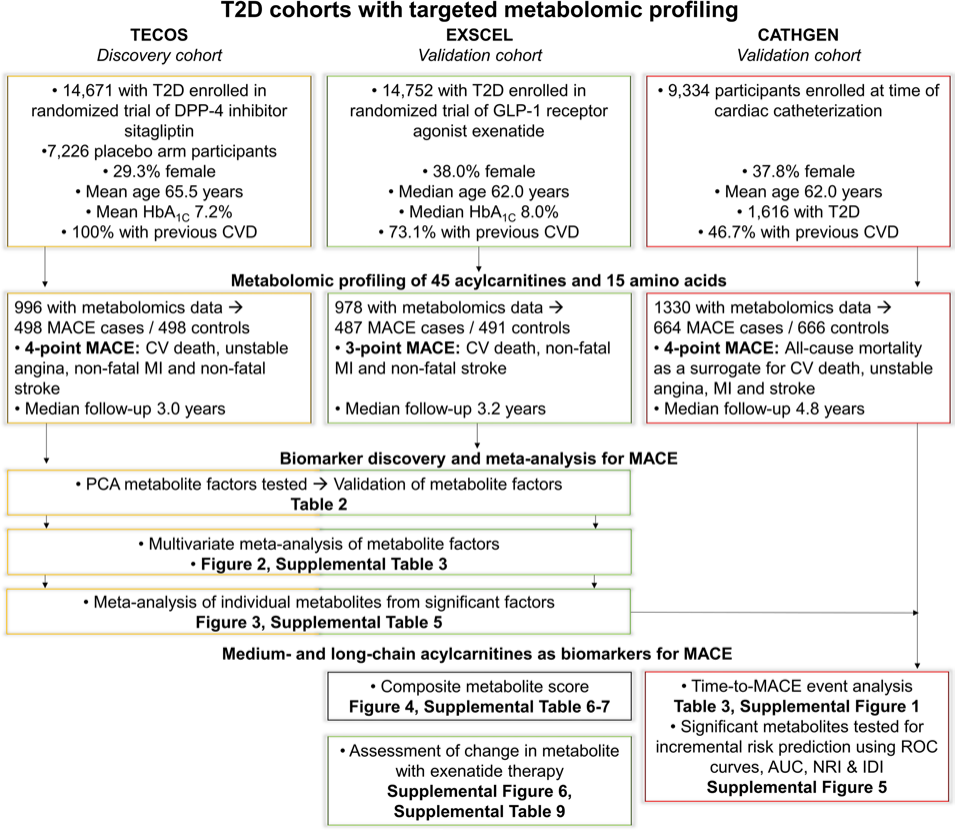
Overview of study design and statistical methods in cohorts with T2D and metabolomic profiling. Previous CVD defined as a history of major CAD, ischemic cerebrovascular disease, or atherosclerotic peripheral arterial disease. IDI, integrated discrimination index.

CV outcome trials have demonstrated that a subset of glucose-lowering medications used for the treatment of T2D also improve CV outcomes, but understanding of the mechanisms of how these medications mitigate risk is limited (15–17). Application of metabolomic profiling may improve biologic understanding and support precision medicine approaches to ultimately improve CV outcomes for patients. Hence, we tested the hypothesis that baseline circulating metabolites reporting on mitochondrial dysfunction predict MACE in individuals with T2D. To accomplish this, we used biospecimens from the Trial Evaluating Cardiovascular Outcomes with Sitagliptin (TECOS) and Exenatide Study of Cardiovascular Event Lowering (EXSCEL), 2 large clinical trial cohorts with systematic capture and confirmation by central blinded adjudication of MACE; and participants with T2D from the Cardiac Catheterization Genetics (CATHGEN) cohort to determine capability of these metabolites for predicting MACE (Figure 1).

## Results

### Baseline participant characteristics.

A nested MACE case-control cohort of 996 participants in the TECOS discovery cohort was examined in the present analyses, including 498 MACE case patients and 498 matched control participants (Table 1). In the EXSCEL validation cohort, a similar nested MACE case-control cohort of 978 participants (*n* = 487 case patients; *n* = 491 control participants) was included. In CATHGEN, 1330 participants with baseline T2D were included. TECOS and EXSCEL participants were older, more often male, and more predominantly of self-reported White race than were participants in the CATHGEN cohort. All 3 cohorts had a high burden of CV risk factors. In TECOS, mean BMI was lower than in EXSCEL and CATHGEN participants. CATHGEN participants had higher systolic BP, higher creatinine levels, and lower prevalence of hypertension, dyslipidemia, CAD, prior myocardial infarction (MI), and prior stroke, but higher prevalence of heart failure (HF). EXSCEL participants had the highest prevalence of peripheral arterial disease and smoking and highest mean hemoglobin A1C level (HbA_1c_). There were no significant differences in baseline cholesterol levels across the 3 cohorts. Baseline participant characteristics stratified by MACE outcome are shown in Supplemental Table 1 (supplemental material available online with this article; https://doi.org/10.1172/jci.insight.168563DS1).

### Medium-chain acylcarnitine metabolites are associated with incident MACE in the TECOS and EXSCEL clinical trials.

Unsupervised machine learning using principal components analysis (PCA) was used to reduce the correlated individual metabolites into 12 orthogonal, uncorrelated metabolite factors or signatures (Supplemental Table 2). Of these, in the TECOS discovery cohort, 1 factor (factor 9), heavily loaded with medium-chain acylcarnitine (MCAC) metabolites, was associated with MACE in univariate models (OR 1.26 [95% CI 1.11–1.45]; for FDR, *P* = 0.008) and multivariate models (OR 1.29 [95% CI 1.11–1.49]; for FDR, *P* = 0.009; Table 2); the OR represents the odds of experiencing an incident MACE event for every 1 SD increase in metabolite-factor levels. This metabolite factor validated for predicting incident MACE in the EXSCEL cohort in univariate analysis (OR 1.15 [95% CI 1.02–1.30]; nominal *P* = 0.03) and multivariate analysis (OR 1.17 [95% CI 1.02–1.34]; *P* = 0.03).

**Figure 2:**
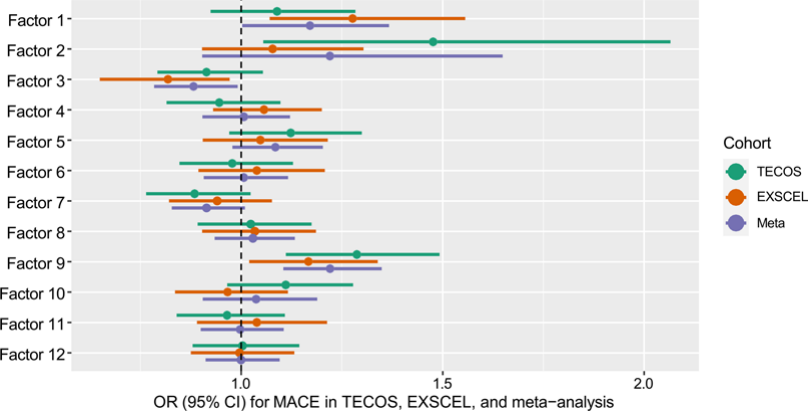
Meta-analysis of PCA metabolite factors for association with MACE in the combined TECOS and EXSCEL cohorts. Forest plot shows multivariate OR for meta-analysis of TECOS and EXSCEL data for association between PCA metabolites factors with MACE. Factor 1 comprises MCACs and LCACs, factor 3 comprises long-chain dicarboxylacylcarnitines, and factor 9 comprises MCACs (*P* < 0.05).

In a post hoc exploratory meta-analysis of multivariate models for all 12 metabolite PCA factors and MACE, 3 PCA factors were nominally associated with MACE: the prior 1 identified through the discovery and validation approach (factor 9 comprises MCACs; OR 1.22 [95% CI 1.11–1.35]; *P* = 9 × 10^–5^) as well as 2 new PCA metabolite factors that did not meet the stringent FDR cutoff for significance in TECOS alone: 1 comprising MCACs and LCACs (factor 1; OR 1.17 [95% CI 1.00–1.37]; *P* = 0.046) and 1 comprising of long-chain dicarboxylacylcarnitines (factor 3; OR 0.88 [95% CI 0.78–0.99]; *P* = 0.04; Figure 2 and Supplemental Table 3).

In exploratory analyses of these 3 metabolite factors with individual components of MACE, there was, overall, a consistent magnitude and direction of effect across the individual outcomes of nonfatal MI, nonfatal stroke, and CV death (Supplemental Table 4), suggesting that these metabolites are associated with risk across heterogeneous MACE events, with the exception of factor 9, which showed strongest effects related to the atherosclerotic outcomes of nonfatal MI and nonfatal stroke.

### Individual metabolites within PCA-derived metabolite factors are also associated with MACE.

**Figure 3:**
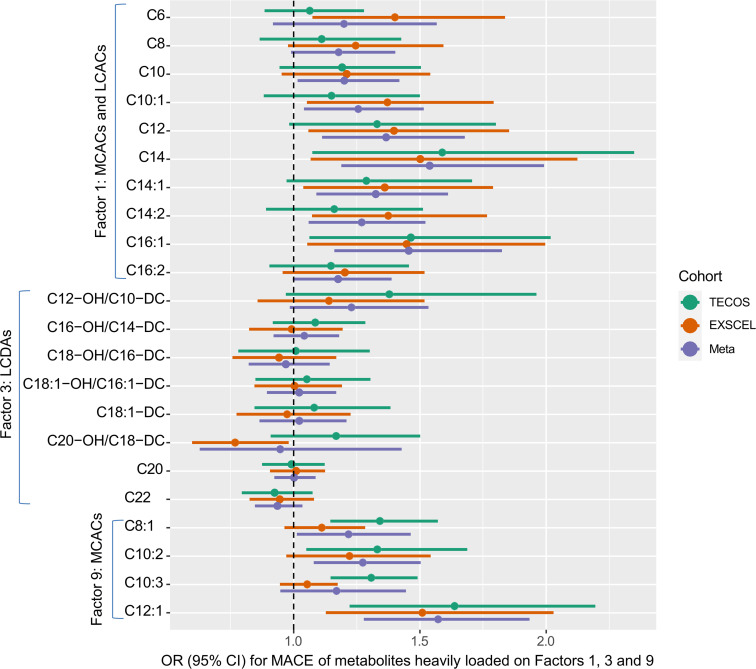
Multivariate models for individual metabolites heavily loaded on PCA factors significantly associated with MACE in meta-analysis. Forest plot shows multivariate meta-analysis of individual metabolites heavily loaded on factors 1, 3, and 9 in TECOS and EXSCEL cohorts. Nine significant metabolites were identified: C8, C10:1, C12-OH/C10/DC, C12:1, C12, C14:1, C14:2, C16:2, and C16:1. LCDA, long-chain dicarboxylacylcarnitine.

To determine the most significant metabolites and evaluate differences in absolute metabolite concentrations, individual metabolites heavily loaded (i.e. absolute value of factor load >0.4) within these 3 significant PCA factors were analyzed for association with incident MACE (*n* = 22 individual metabolites) in meta-analyses combining TECOS and EXSCEL data. In univariate meta-analyses, 12 individual metabolites were associated with MACE, all with higher levels in MACE cases compared with non-MACE controls: MCACs and LCACs (all even-chain acylcarnitines, ranging from 8 to 16 carbons in length) and 1 medium-chain dicarboxylacylcarnitine (C12-OH/C10-DC) (OR range 1.17–1.66; *P* < 0.05; Supplemental Table 5). In multivariate meta-analyses, 10 MCACs and LCACs remained significant (OR range 1.20–1.57; *P* < 0.05; Figure 3 and Supplemental Table 5). The OR represents the odds of experiencing an incident MACE event for every 1 unit increase in the log-transformed metabolite level. These results suggest that a single metabolite is not driving the association of the metabolite factors with MACE but, instead, that multiple correlated metabolites are associated individually and as a group.

### Metabolites are associated with time to MACE and improve prediction of clinical outcomes in individuals with T2D.

Given the nested case-control design of the TECOS and EXSCEL cohorts, we analyzed a third cohort (*n* = 1330 participants with T2D from the CATHGEN cohort) to enable time-to-event and incremental risk prediction analyses (Table 1). Of these, 664 individuals experienced MI, unstable angina, stroke, or all-cause mortality in the CATHGEN study with a median time to event of 509 days (IQR 172–997 days). All 13 unique metabolites identified in either univariate or multivariate TECOS and EXSCEL meta-analysis were associated with time to MACE in the CATHGEN group in univariate models (*P* < 0.05); 9 of these metabolites (MCACs, LCACs, and 1 dicarboxylacylcarnitine) remained significant in multivariate models (*P* < 0.05; Table 3). The HR represents the odds of experiencing an incident MACE event for every 1 unit increase in the log-transformed metabolite level. Kaplan-Meier curves by tertile of metabolite are shown in Supplemental Figure 1. The significance of the 4 metabolites that did not remain significant in multivariate models (MCACs and LCACs) were attenuated primarily by age, history of HF, and creatinine level.

**Figure 4:**
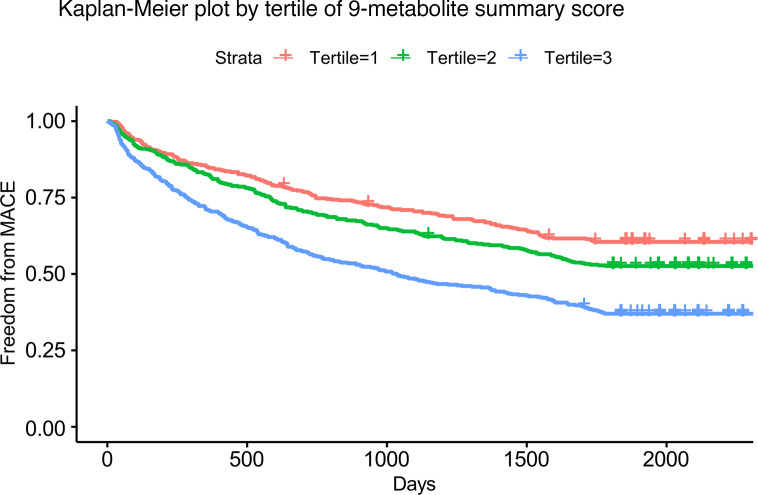
Kaplan-Meier curve for MACE in CATHGEN by tertile of 9-metabolite score (C8, C10:1, C12-OH/C10/DC, C12:1, C12, C14:1, C14:2, C16:2, C16:1). Events were defined starting at 30 days after date of index catheterization and study enrollment to avoid procedural related events. A total of 664 individuals in the CATHGEN cohort experienced MACE, with a median time to event of 509 days.

Having converged on these 9 metabolites as the most significant individual metabolites independently associated with MACE and time to MACE in multivariate models in 3 cohorts, we then created a composite score of the 9 metabolites by log-transforming the molar sum of these metabolites and then scaling this value. Supplemental Figure 2 demonstrates absolute values of the individual metabolites and Supplemental Figure 3 demonstrates the log-transformed, scaled sum of the 9-metabolite score stratified by MACE in the 3 cohorts. Analyzed as a continuous variable in TECOS and EXSCEL, this composite metabolite score was associated with MACE in multivariate meta-analysis models (OR 1.16 [95% CI 1.06–1.29]; *P* = 0.002; Supplemental Table 6 and Supplemental Figure 4). To directly compare the effect size with the individual metabolites in a meaningful way, we analyzed the 9 individual metabolites again after scaling their log-transformed levels. The range of the HR in multivariate meta-analysis models was 1.09–1.25. In the CATHGEN cohort, the composite 9-metabolite score was also associated with time to MACE in continuous analyses (multivariate HR 1.26 [95% CI 1.15–1.37]; *P* = 1.5 × 10^–7^; Supplemental Table 7). For comparison, the multivariate HR range of the scaled 9 individual metabolites was 1.13–1.25. Kaplan-Meier curves by tertile of the metabolite score are shown in Figure 4. The composite score showed relative similarly magnitude of effect sizes across MACE subcomponents, with perhaps slightly greater risk associated with CV death and lesser magnitude of risk for nonfatal MI (Supplemental Table 8).

We then assessed whether these 9 metabolites demonstrated incremental risk prediction, using receiver operator characteristic (ROC) curves. In the CATHGEN cohort, the addition of these 9 metabolites was statistically significant but had minimal effect on improving the discriminative capability for MACE over the clinical model alone (i.e., age, sex, race, history of HF, CAD, BMI, HbA_1c_, systolic BP, creatinine and LDL cholesterol [LDL-C] levels, and smoking status): AUC = 0.71 (95% CI 0.68–0.74) versus 0.70 (95% CI 0.67–0.73); *P* = 0.03 by DeLong’s test (Supplemental Figure 5). In reclassification analyses comparing addition of the 9 individual metabolites with the clinical model alone, the overall net reclassification index (NRI) was 0.24 (*P* = 2.4 × 10^–5^), the net proportion of case patients assigned to a higher-risk group in the model inclusive of metabolites was 7.1% (*P* = 0.07), and the net proportion of control participants assigned to a lower-risk group was 16.5% (*P* = 3.3 × 10^–5^). The integrated discrimination index, which represents the improvement of the slopes of discrimination curves between the old and new models, was 0.014 (*P* = 1.9 × 10^–5^).

### Pharmacologic therapy with exenatide beneficially modifies metabolite levels in individuals with T2D.

Levels of 7 of the 9 tested metabolites significantly changed between baseline and 12-month samples in individuals randomized to once-weekly exenatide compared with placebo (nominal *P* < 0.05). These included mean levels of LCACs that decreased with exenatide therapy and increased in participants randomly assigned to receive placebo randomized participants (Supplemental Figure 6 and Supplemental Table 9), suggesting a beneficial effect of exenatide on these metabolites. MCAC and dicarboxylacylcarnitine levels increased to a lesser extent with exenatide therapy compared with placebo. Changes in these metabolites with exenatide remained significant even after adjustment for the amount of weight loss (a known effect of glucagon-like peptide-1 receptor agonist [GLP-1 RA]; Supplemental Table 9). Similar changes were seen for exenatide therapy as compared with placebo for the composite 9-metabolite score (*P* = 0.01; Supplemental Figure 6), and there was no change in the results after adjusting for amount of weight loss. Correlation between the change in the 9-metabolite summary score and change in weight loss revealed a moderate positive correlation (Pearson’s ρ, *P* = 0.49).

## Discussion

Leveraging 2 large, international, randomized clinical trials of glucose-lowering medications for T2D, we report, in one of the largest studies of its kind, higher baseline levels of 9 metabolites that reflect dysregulated mitochondrial fatty acid oxidation in individuals with T2D who subsequently experienced MACE compared with controls without subsequent MACE. These results support the potential clinical utility of these metabolites as biomarkers for risk prediction, given that results were robust to adjustment for covariables and were beneficially modified by exenatide. These mitochondrial metabolites, consisting primarily of MCACs and LCACs, may reflect subclinical impairments in mitochondrial fatty acid oxidation, identifying important molecular signatures for incident MACE in individuals with T2D. These results highlight potential metabolic biomarkers for MACE risk prediction and also highlight potential mechanisms for the beneficial effects of this class of GLP-1 RA medications, which are being increasingly used for glycemic control and weight loss.

**Figure 5:**
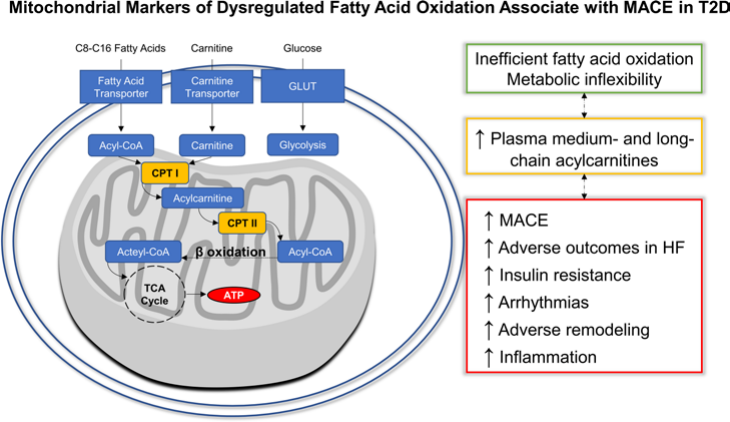
Mitochondrial markers of dysregulated fatty acid oxidation associate with MACE in T2D. Medium- and long-chain fatty acids are transported across the plasma membrane by fatty acid transporters and then converted into their acyl-CoA and cross the outer mitochondrial membranes. The inner mitochondrial membrane is impermeable to acyl-CoAs and carnitine palmitoyl transferase I is required to esterify the acyl-CoA plus carnitine into acylcarnitines. Inside the inner mitochondrial membrane, carnitine acyltransferase is reverse esterified back to an acyl-CoA to undergo β oxidation with carbon chain removal until an acetyl-CoA remains to enter the TCA cycle and, ultimately, the electron transport chain for ATP energy production. In states of impaired mitochondrial fatty acid β oxidation and metabolic inflexibility, plasma levels of MCACs and LCACs levels may increase. Based on the present analyses in T2D, increased levels of MCACs and LCACs associate with incident MACE. The working hypothesis for the data presented here is that inefficient fatty acid oxidation in the mitochondria contribute to the bottle neck of substrate utilization and buildup of circulating levels of acylcarnitines. However, the tissue source is not clear and, alternatively, accumulations in these metabolites could also reflect increased mitochondrial fatty acid flux with incomplete fatty acid oxidation. CPT I, carnitine palmitoyltransferase I; CPT II, carnitine palmitoyltransferase II.

Mitochondrial metabolism is central to cardiac and skeletal muscle function (Figure 5). In healthy cardiac metabolism, mitochondrial oxidative phosphorylation of fatty acids supplies the majority of ATP, followed by carbohydrates via glycolysis (18). In a normal, healthy state, myocardial metabolism has broad flexibility, with the ability to use a range of substrates for efficient energy production. However, perturbations in energy metabolism in T2D and CVD can contribute to worsened CV function and adverse clinical outcomes (19). In T2D, levels of circulating free fatty acids, triglycerides, and LCACs are elevated, and preclinical models have shown accumulation of LCACs in skeletal muscle, representing impaired mitochondrial fatty acid β oxidation (12). There is also increased myocardial fatty acid uptake in T2D, altering substrate supply and impairing fatty acid β oxidation. During ischemia, glycolysis becomes a main source of energy production, but the duration for which this can be maintained is limited and can ultimately lead to impaired contractile function secondary to intracellular buildup of acids and ions (20). Similarly, in HF, decreased mitochondrial oxidative capacity results in a compensatory increase in glucose uptake for glycolysis and overall metabolic inflexibility with increased reliance on alternative substrate utilization (21).

Eight of the 9 metabolites that associated with MACE in the TECOS, EXSCEL, and CATHGEN cohorts are MCACs and LCACs, which report on impaired mitochondrial fatty acid β oxidation. Rare mitochondrial disorders of lipid metabolism caused by deficiencies in carnitine transfer enzymes are associated with skeletal and cardiac myopathy and also display elevated levels of these metabolites (22). Acylcarnitines accumulate as a result of inefficient fatty acid oxidation from either enzymatic defects or imbalances in fatty acid oxidation to TCA flux creating a bottleneck of carbon substrates (12, 23). LCACs are associated with insulin resistance because chronic overnutrition can lead to lipotoxicity and interference with insulin signaling via multiple mechanisms, including incomplete β oxidation at the mitochondrial membrane (12–14, 24). One additional metabolite associated with MACE in all 3 cohorts was a dicarboxylacylcarnitine metabolite; these metabolites may similarly reflect changes in β oxidation or may indicate changes in ER carboxylation via microsomal P450 or peroxisomal metabolism (25). The composite 9-metabolite score was not stronger than the individual metabolite analyses; this is likely related to the strong correlation of biologically grouped metabolites. When applying these metabolites to clinical prediction tools, the AUC significantly improved, though only minimally, suggesting that these metabolites report on important biology but may not improve discrimination over a clinical model alone. However, these metabolites do appear to improve down reclassification of risk (NRI), suggesting they may have some clinical utility.

Here, in 1 of the largest such studies, we extend and refine prior studies with a focus on participants with T2D, evaluating MACE and its subcomponents as well as changes with pharmacologic therapy. In previous work with 2023 CATHGEN participants, MCACs were identified as predictors of all-cause mortality (8). In the present study, we find overall consistent effect sizes across MACE subcomponents; however, factor 9, which comprises MCACs, may be more strongly related to atherosclerotic disease given its strongest associations were with nonfatal MI and nonfatal stroke. In 4164 participants with suspected angina who were recruited at University Hospitals in Norway, MCACs and LCACs were associated with CV death and acute MI with no effect modification according to T2D or BMI (26). These metabolites have also been associated specifically with cardioembolic stroke and stroke recurrence in the Korea University Stroke Registry and correlate with other stroke risk factors, including age, atrial fibrillation, hypertension, and male sex (27). In the context of cardiometabolic disease, higher levels of LCACs have been identified in obese and insulin-resistant individuals compared with lean control participants (11). Plasma levels of LCACs are higher in HF, are higher in end-stage HF versus chronic stable HF, and are also differentially elevated in HF with reduced versus preserved ejection fraction (28, 29). In a study of 1032 participants in the Henry Ford Heart Failure Pharmacogenomic registry, MCACs and LCACs associated with ischemic etiology of HF and a prognostic metabolite profile comprising 13 metabolites, including C18:1, added incremental risk prediction for survival over N-terminal pro-B-type natriuretic peptide (NT-proBNP) over a median follow-up of almost 3 years (30). Similarly, in the Heart Failure: a Controlled Trial Investigating Outcomes of Exercise Training (HF-ACTION), a randomized controlled trial of exercise training in ambulatory patients with HF, metabolomic profiling of 664 participants identified LCACs as associated with impaired cardiorespiratory fitness (measured by peak oxygen consumption) and adverse clinical outcomes, including death and hospitalization (31).

Prior studies have suggested that these metabolites are modifiable by exercise and pharmacologic therapy. For example, in the HF-ACTION trial, LCACs levels were found to be beneficially modifiable with exercise (i.e., they decreased), but in analyses stratified by T2D, these metabolites decreased to a lesser extent with exercise in patients with T2D (32). Furthermore, stronger associations of these metabolites with death and hospitalization were seen in participants with T2D compared with those without T2D (32). Interestingly, metabolomic profiling of 234 participants in the Dapagliflozin Effects on Biomarkers, Symptoms and Functional Status in Patients with HF with Reduced Ejection Fraction (DEFINE-HF) trial, in which patients with HF with reduced ejection fraction were randomly assigned to receive 12 weeks of the sodium-glucose co-transporter-2 inhibitor (SGLT2i) dapagliflozin or placebo, found that MCACs and ketone-related metabolites increased with SGTL2i (33). Increases in short-chain acylcarnitines and MCACs without an increase in LCACs may suggest an overall increased in fatty acid oxidation secondary to metabolic reprogramming from SGLT2i therapy. In DEFINE-HF participants, increases in LCACs and dicarboxylacylcarnitines were associated with intermediate outcomes, including quality of life and NT-proBNP, regardless of SGLT2i therapy. In exploratory analyses presented here, we found that MCACs, LCACs, and dicarboxylacylcarnitines were favorably modified by exenatide, that is, their levels increased to a lesser extent in individuals randomized to the exenatide group as compared with the placebo group (MCACs and dicarboxylacylcarnitine) or decreased in those randomized to receive exenatide while increasing in individuals receiving placebo (LCACs). Importantly, change in weight loss with exenatide therapy did not fully explain this association, suggesting alternative direct or indirect effects of exenatide therapy. GLP-1 RAs have gained FDA approval and increased clinical utility for glycemic control and weight management; the metabolic findings here may identify mechanisms of beneficial class effects that could be translated to future patient care. Given that these metabolites are modifiable by lifestyle and pharmacologic interventions, if they are ultimately demonstrated to be in causal pathways, they could identify potential pharmacologic targets and possibly identify individuals for personalized therapies.

There are several strengths to our approach. First, we used samples from 2 robust clinical trials of CV outcomes of participants with T2D, with centrally adjudicated outcomes blinded by treatment assignment as well as a third validation cohort of participants with T2D undergoing evaluation for ischemic heart disease. Many biologically relevant metabolites were accurately measured with the addition of internal standards enabling absolute quantification. We used a discovery approach with careful adjustment for multiple comparisons of metabolites factors and inclusion of 2 validation cohorts. Finally, we applied these findings to demonstrate their potential clinical utility by assessing incremental risk-predictive capabilities and showed that they are modified by GLP-1 RA therapy.

Important study limitations should be noted, however. Because the majority of participants in these studies have obesity and all have T2D, differential findings in levels of these metabolites and impact on MACE outcomes or treatment effects by BMI and diabetes status could not be thoroughly assessed. Nonalcoholic fatty liver disease is another relevant comorbid condition that has reported effects on MACE and is likely to be present in these populations, but this was unable to be determined in these cohorts. Importantly, we cannot determine the tissue source of MCACs and LCACs in this study, which could include myocardial or skeletal origin. The liver is not a high-energy consumptive organ (compared with ATP use in cardiac and skeletal muscle) and, therefore, is not known to be a source of circulating acylcarnitine levels. Though we hypothesize these findings are due to inefficient fatty acid oxidation, increases in TCA flux or changes in enzymatic expression could also be contributing; these were not able to be analyzed here. Furthermore, although we conducted careful multivariate adjustments, other emerging biomarkers of MACE risk were not assessed. Finally, although we adjusted for a measure of glycemia (HbA_1c_), we were not able to adjust for other potential clinical risk factors including insulin resistance, physical activity, or diet.

In 3 cohorts of individuals with T2D, with a total of 1649 MACE events analyzed, we demonstrate herein the power of applying robust metabolomic profiling technologies to clinical trials of T2D glucose-lowering medications to identify relevant biology and potential clinical utility of related circulating biomarkers reporting on dysregulated mitochondrial metabolism. Because all participants in the present study had T2D and we adjusted for HbA_1c_ levels, these findings suggest additional potential influence of impaired mitochondrial efficiency at the molecular level that associates with future CV events. Extending prior observations, the present results confirm associations between selected metabolites reflecting mitochondrial dysfunction and risk for atherosclerotic and thrombotic CVD complications, adding to literature describing the importance of acylcarnitines in CVD and, furthermore, highlight potential mechanisms of effect of GLP-1 RA medications. Incorporation of measurement of these metabolites for individuals with T2D may aid in risk stratification and, ultimately, identification of novel therapeutic targets to limit the burden of MACE in this population.

Taken together, elevated levels of MCACs and LCACs may reflect abnormalities in biologic pathways of energy use in either myocardium, peripheral skeletal muscle, or both. In the present work, higher levels of MCACs and LCACs in individuals with T2D may reflect subclinical metabolic inflexibility and inefficient mitochondrial fatty acid oxidation but are modifiable by GLP-1 RA therapy. Further work is warranted to determine the clinical applicability of these findings to CV risk prediction and the role of these biomarkers in specific types of CVD, as well as to evaluate whether these findings are generalizable to other GLP-1 RA medications.

## Methods

### Study populations

#### TECOS clinical trial biomarker substudy.

The discovery cohort consisted of participants from the placebo arm of TECOS (34). TECOS included 14,671 participants in total, with 7226 in the placebo arm. Briefly, TECOS was a randomized, placebo-controlled CV-outcomes trial of sitagliptin, a dipeptidyl peptidase 4 inhibitor, in individuals with T2D and established atherosclerotic CVD. Randomization occurred between December 2008 and July 2012, and the trial concluded in March 2015. Participants at baseline were at least 50 years of age with an HbA_1c_ between 6.5% and 8.0%; they were followed for a median of 3.0 years. The primary outcome was time to the first event of the composite of unstable angina, nonfatal MI, nonfatal stroke, or CV death. A nested, matched MACE case-control subset of all TECOS placebo-arm participants with available baseline peripheral blood samples who experienced incident MACE were identified (*n* = 498) and matched 1:1 with control participants who were selected from the placebo group and matched on history of HF, CAD, BMI, HbA_1c_, creatinine and LDL-C levels, fasting status, and left ventricular ejection fraction.

#### EXSCEL clinical trial biomarker substudy.

The validation cohort consisted of participants from EXSCEL, a randomized, placebo-controlled trial of once-weekly exenatide, a GLP-1 RA (35). Overall, EXSCEL included 14,752 adult participants with T2D, with HbA_1c_ levels between 6.5% and 10.0%, approximately 70% of whom had CVD. Randomization occurred between June 2010 and September 2015. The trial concluded in May 2017; participants were followed for a median of 3.2 years. The EXSCEL primary outcome was time to the first event of the composite of nonfatal MI, nonfatal stroke, or CV death (MACE). Overall, 978 participants (*n* = 487 case patients and 491 control participants) were identified and their data were used for metabolomic profiling in the present analyses.

#### CATHGEN cohort.

The CATHGEN study includes 9334 patients who underwent cardiac catheterization at Duke University Medical Center (Durham, NC) between January 2001 and December 2010 (36). For the present analyses, 1330 participants with a diagnosis of T2D at the time of study enrollment and available metabolomics data were included. Demographic and comorbidity data were collected through medical record review at study enrollment, and yearly follow-up was conducted for events and vital status. These data were supplemented with review of electronic health records. Clinical outcomes were determined using *International Classification of Diseases, Ninth Revision* and *Tenth Revision* codes at least 30 days from study enrollment and Social Security Death Index and National Death Index data. A composite outcome of unstable angina, nonfatal MI, nonfatal stroke, and all-cause mortality (MACE) was defined and used in time-to-event analyses. To exclude procedural-related events, incident events were defined starting at 30 days after date of index catheterization and study enrollment.

### Metabolomic profiling

Tandem flow-injection mass spectrometry was used to quantify 60 metabolites (*n* = 45 acylcarnitines and 15 amino acids) in frozen plasma samples that were previously unthawed. Samples were collected under a standardized study protocol. Baseline metabolites measured in 996 TECOS samples, 978 EXSCEL samples, and 1330 CATHGEN samples were used in the present analyses. As described previously, proteins were removed by precipitation (6). Acylcarnitines and amino acids were esterified with hot acidic methanol and *n*-butanol, respectively. Mass spectrometry was performed with a Xevo TQD instrument (Waters Corp.). Internal standards were added to enable quantitative assessment of metabolites (4); CVs have been previously reported (6). Metabolomic assays were performed by the Metabolomics Core Laboratory at the Duke Molecular Physiology Institute. Staff at the Core Laboratory were blinded to clinical characteristics and outcomes of participant samples.

### Statistics

Metabolites with greater than 25% of values below lower limits of detection (LOD) were excluded from analyses (1 metabolite: C7-DC acylcarnitine); metabolite values below LOD were analyzed as 0. Given high collinearity between metabolites residing in shared biologic pathways, PCA with varimax rotation was used for dimensionality reduction in the TECOS discovery cohort, resulting in orthogonal factors composed of a weighted sum of correlated metabolites. PCA factors with an eigenvalue greater than 1 were retained (Kaiser criterion). PCA weights for each metabolite within each factor as created in the TECOS study population were projected onto each individual in the EXSCEL cohort to create the same metabolite factors. All metabolites were log-transformed prior to analyses.

For the matched sample set in the TECOS discovery cohort, conditional logistic regression of PCA factors was used to test association with MACE events in both univariate and multivariate models using FDR *P* < 0.1. Multivariate models in TECOS included relevant covariates for which participants were not matched: age, sex, race, systolic BP, and smoking status. PCA metabolite factors significant after adjustment for multiple comparisons in TECOS were then assessed for association with MACE in the EXSCEL validation cohort, using both univariate and multivariate logistic regression; nominal validation was considered at *P* < 0.05. Multivariate models in EXSCEL were adjusted for age, sex, race, history of HF, CAD, BMI, HbA_1c_, systolic BP, creatinine and LDL-C levels, and smoking status. Median imputation was used for missing LDL-C values.

Post hoc exploratory meta-analyses of multivariate models from TECOS and EXSCEL were then performed using the *meta* package in R; metabolite factors with nominal *P* < 0.05 were identified. Analyses were also conducted for subcomponents of MACE (namely, nonfatal MI, nonfatal stroke, and CV death) and meta-analyzed. Sensitivity analyses of individual metabolites within PCA factors significant in meta-analyses with an absolute factor loading (>0.4) was also performed using meta-analyses to determine the most important individual metabolites (*P* < 0.05).

Given that the case-control study design of the TECOS and EXSCEL cohorts precluded time-to-event analyses, we used the CATHGEN cohort data to determine if identified significant individual metabolites from the TECOS and EXSCEL analyses were associated with time to MACE. Specifically, we used an accelerated failure time (AFT) parametric model based on a Weibull distribution to estimate the relationship between time to MACE event and significant metabolites. We used ROC curves with AUC analyses to assess the incremental value of metabolites significant in the AFT analyses to predict MACE on top of a clinical model (age, sex, race, history of HF, CAD, BMI, HbA_1c_, systolic BP, creatinine and LDL-C levels, and smoking status). NRI and integrated discrimination improvement analyses were used to evaluate the improvement in prognostic discrimination after adding significant metabolites (37, 38). Median imputation was used for missing HbA_1c_, creatinine, and LDL-C values. Significance was considered at *P* < 0.05.

A composite score was then created from significant metabolites. Given the variable magnitude in absolute value of metabolites, metabolites were summed, log-transformed, and then scaled for analyses. In CATHGEN, C12-OH/C10-DC had 15% over values below lower limits of quantification; these values were median imputed for composite metabolite score calculations. This composite sum was then tested in univariate and multivariate models for MACE using conditional logistic regression followed by meta-analysis in TECOS and EXSCEL and using AFT models in CATHGEN. Composite metabolite scores were tested as continuous variables in each cohort. The composite metabolite score was also used to test outcomes in the MACE subcomponents nonfatal MI, nonstroke, and CV death in TECOS and EXCSEL and meta-analyzed with the score treated as a continuous variable.

To assess whether metabolite levels can be modified by GLP-1 RA, we tested the change in metabolite levels between baseline and 12-month plasma samples from 973 EXSCEL participants, using Mann-Whitney-Wilcoxon tests for significant individual metabolites. Only baseline biospecimens from participants in the placebo arm of the TECOS trial were available for the present analyses; therefore, change in metabolite level with sitagliptin could not be tested. For these exploratory analyses, significance was considered at nominal *P* < 0.05. To determine whether change in metabolite with exenatide was explained by weight loss with exenatide, we performed a linear mixed model with random intercepts for participants, including an interaction term between time point and treatment, and adjusting for change in weight. The composite metabolite score was also used to test for interactions with exenatide treatment using linear mixed models. Finally, the correlation between change in metabolite score and change in weight was tested.

### Study approval

All study participants in TECOS, EXSCEL, and CATHGEN gave written informed consent for participation in the parent study and for use of their stored biospecimens for future use. The IRB for each site approved the primary studies and the Duke IRB approved this biomarker substudy.

### Data availability

The data sets generated for these analyses will be shared on reasonable request to the corresponding author for review by the EXSCEL and TECOS publications committees and the CATHGEN committee.

## Author contributions

JAR, RJM, MN, JBG, LKT, PA, EDP, AFH, RRH, and SHS designed the study. OI and CBN performed and oversaw metabolomics measurements. MN performed statistical analyses. RJM, JBG, JBB, HS, CDS, NS, RWM, YZ, DKM, ES, PA, EDP, AFH, RRH, and SHS provided expertise and oversight for TECOS and EXSCEL trial data. SHS provided expertise and oversight for CATHGEN data. JAR, MN, and SHS wrote the manuscript; RJM, JBG, LKT, OI, CBN, JBB, HS, CDS, NS, RWM, YZ, DKM, ES, PA, EDP, AFH, and RRH provided critical review and edits.

## Supplementary Material

Supplemental data

Supplemental tables 1-8

Supporting data values

## Figures and Tables

**Table 1:**
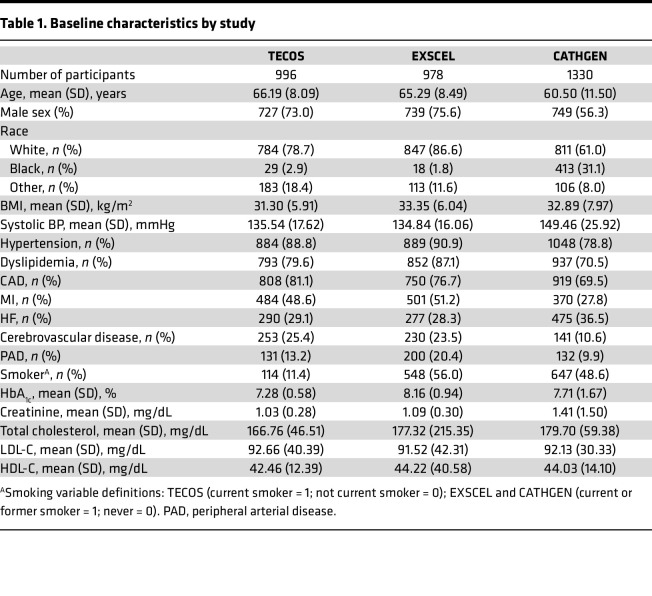
Baseline characteristics by study

**Table 2:**
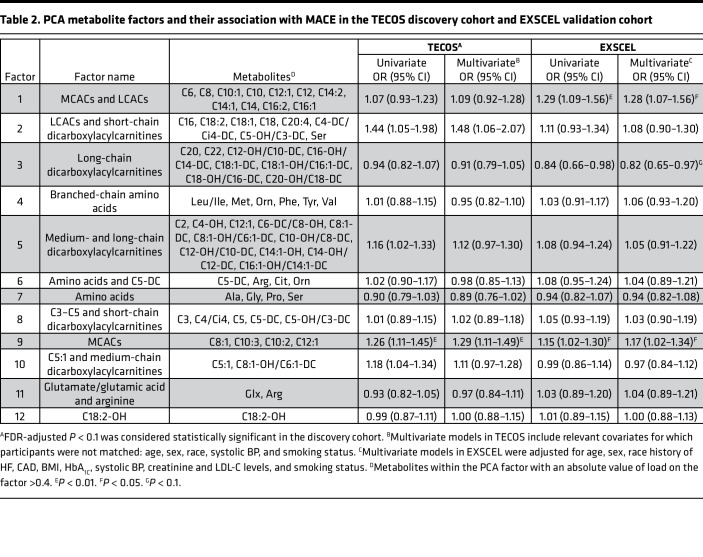
PCA metabolite factors and their association with MACE in the TECOS discovery cohort and EXSCEL validation cohort

**Table 3:**
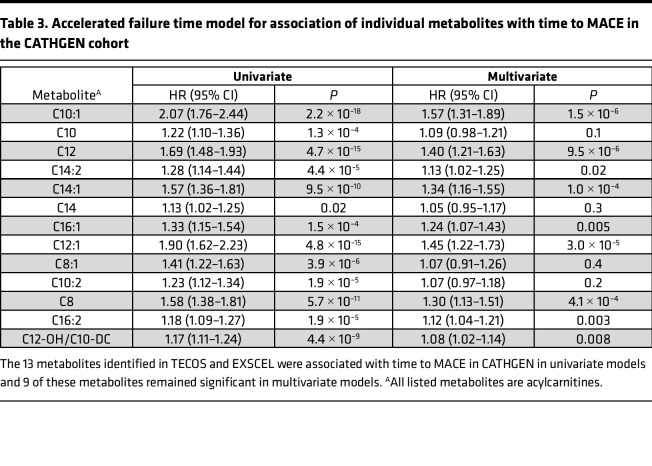
Accelerated failure time model for association of individual metabolites with time to MACE in the CATHGEN cohort

## Version 1 08/08/2023

In-Press Preview

## Version 2 09/08/2023

Electronic publication
