# Supplementary material for: Mitochondrial metabolites predict adverse cardiovascular events in individuals with diabetes
Source: JCI Insight. 2023 Sep 8;8(17):e168563. doi: 10.1172/jci.insight.168563 (PMC10544215; doi:10.1172/jci.insight.168563)

Supplemental Figure 1. Kaplan-Meier curves for metabolites significantly associated with MACE in CATHGEN

Kaplan-Meier curve for MACE in CATHGEN (N=1330) by tertile of metabolite for each of the nine individual significant metabolites. Events were defined starting at 30 days after date of index catherization and study enrollment to avoid procedural related events. 664 individuals suffered MACE in CATHGEN with a median time-to-event of 509 days.

A. Kaplan−Meier plot by tertile of C10:1

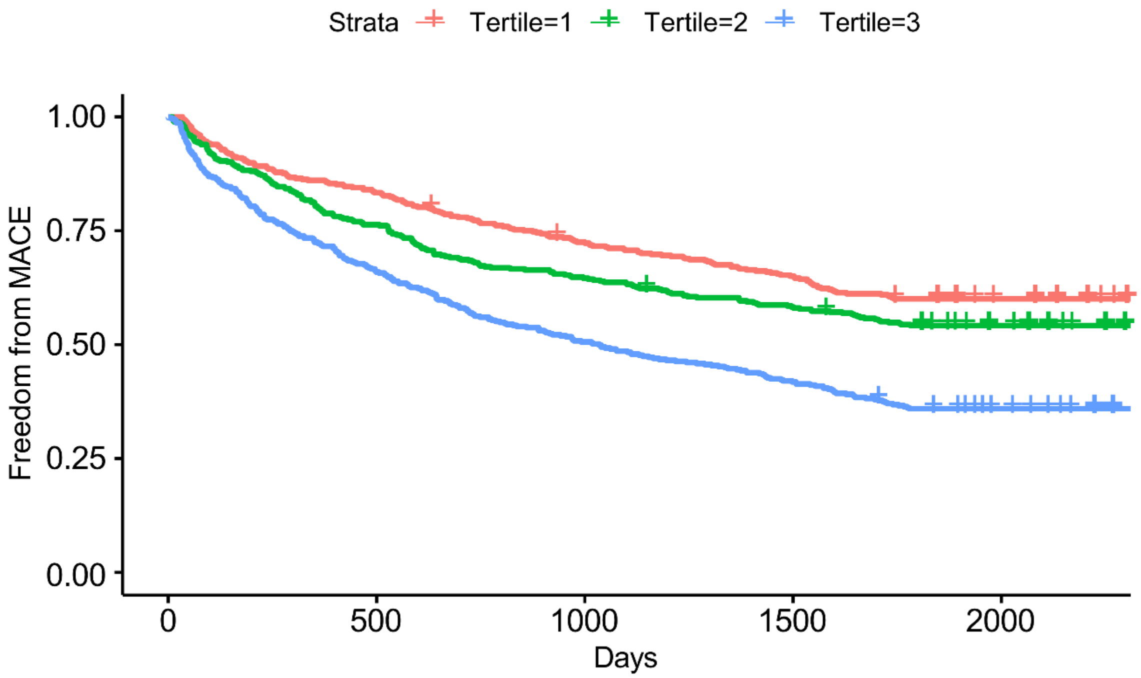

B. Kaplan−Meier plot by tertile of C12

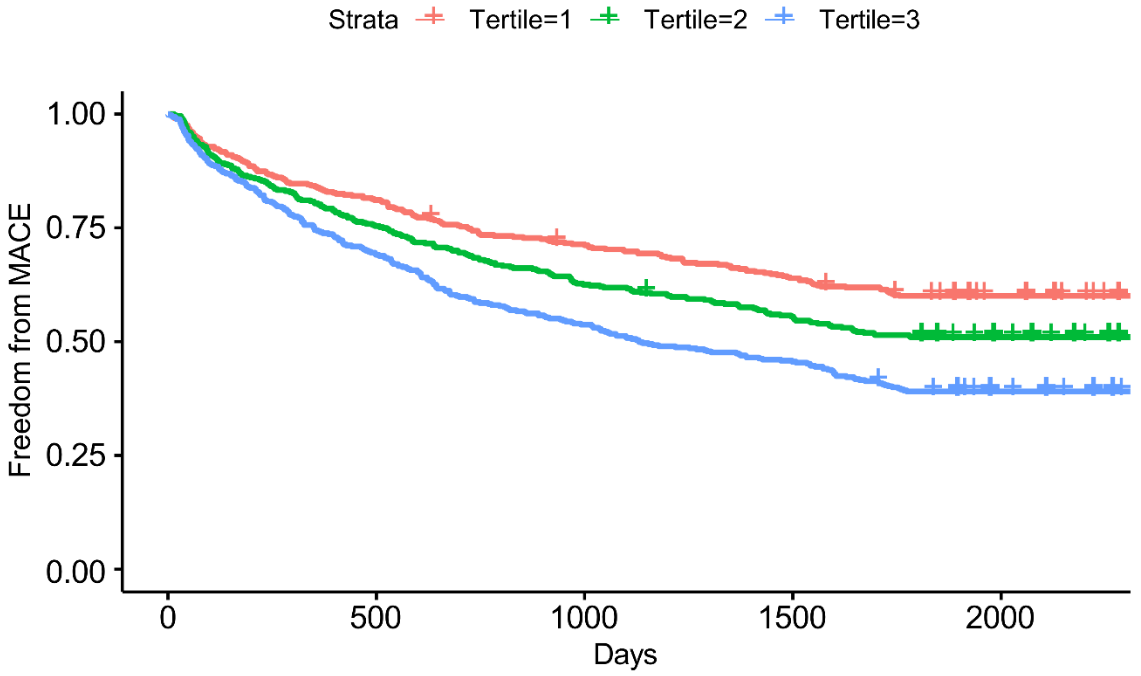

C. Kaplan−Meier plot by tertile of C14:2

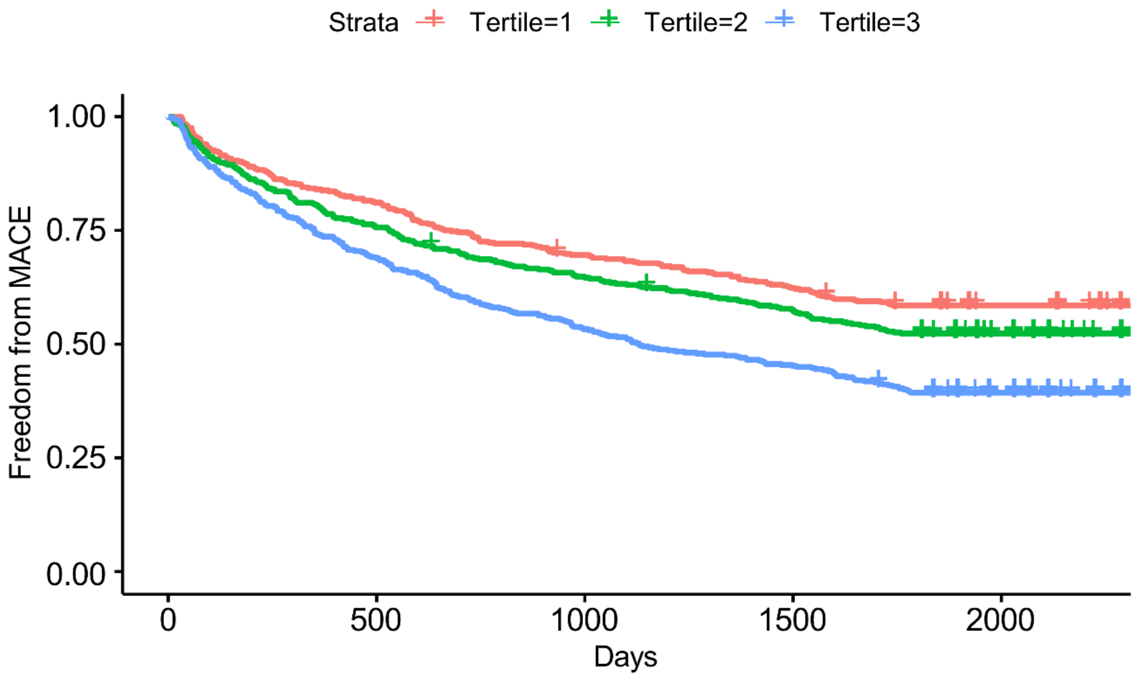

D. Kaplan−Meier plot by tertile of C14:1

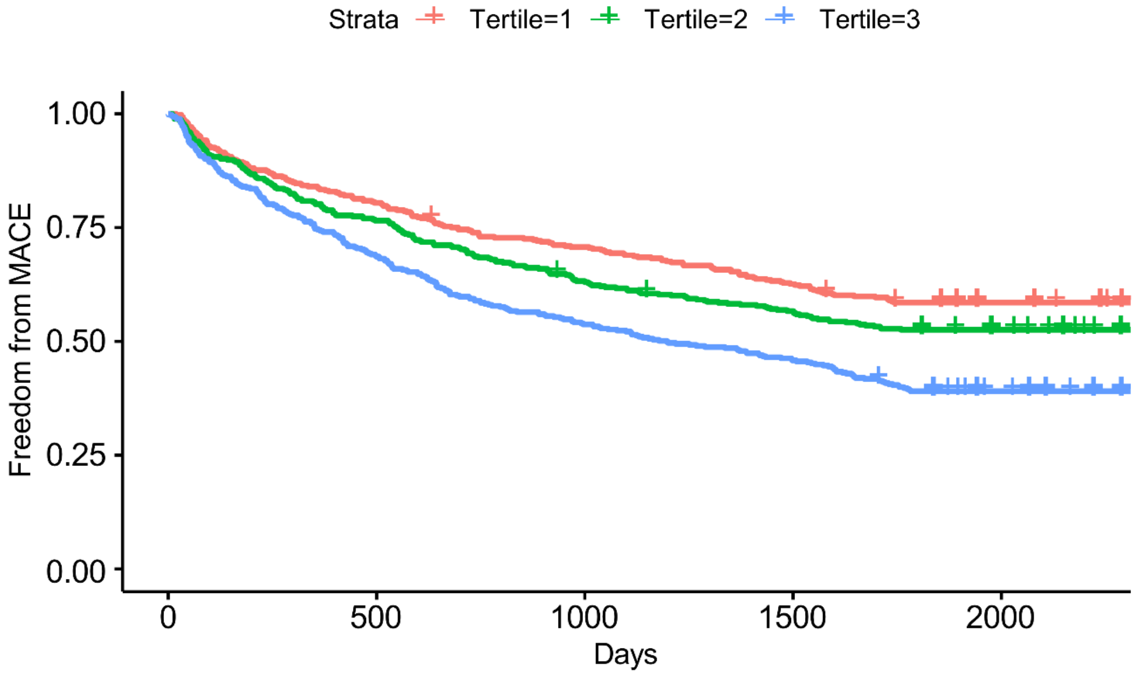

E.

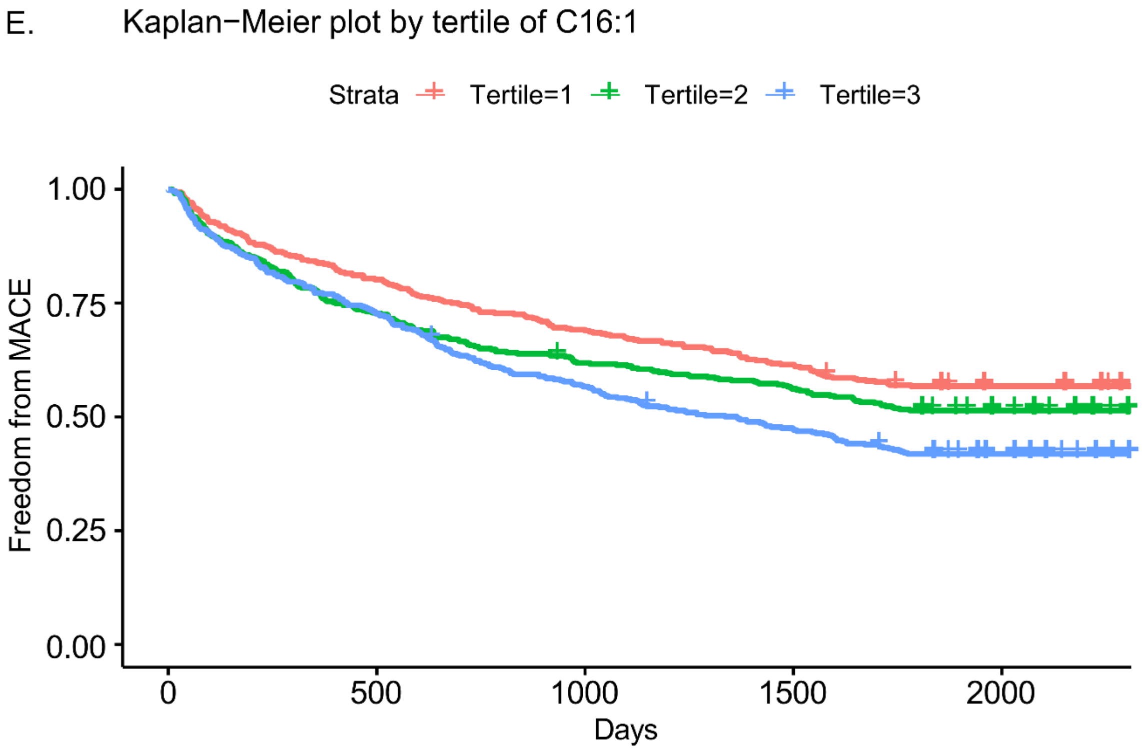
Kaplan−Meier plot by tertile of C16:1
Strata
Tertile=1
Tertile=2
Tertile=3
1.00
0.75
0.50
0.25
0.00
Freedom from MACE
0
500
1000
1500
2000
Days

F.

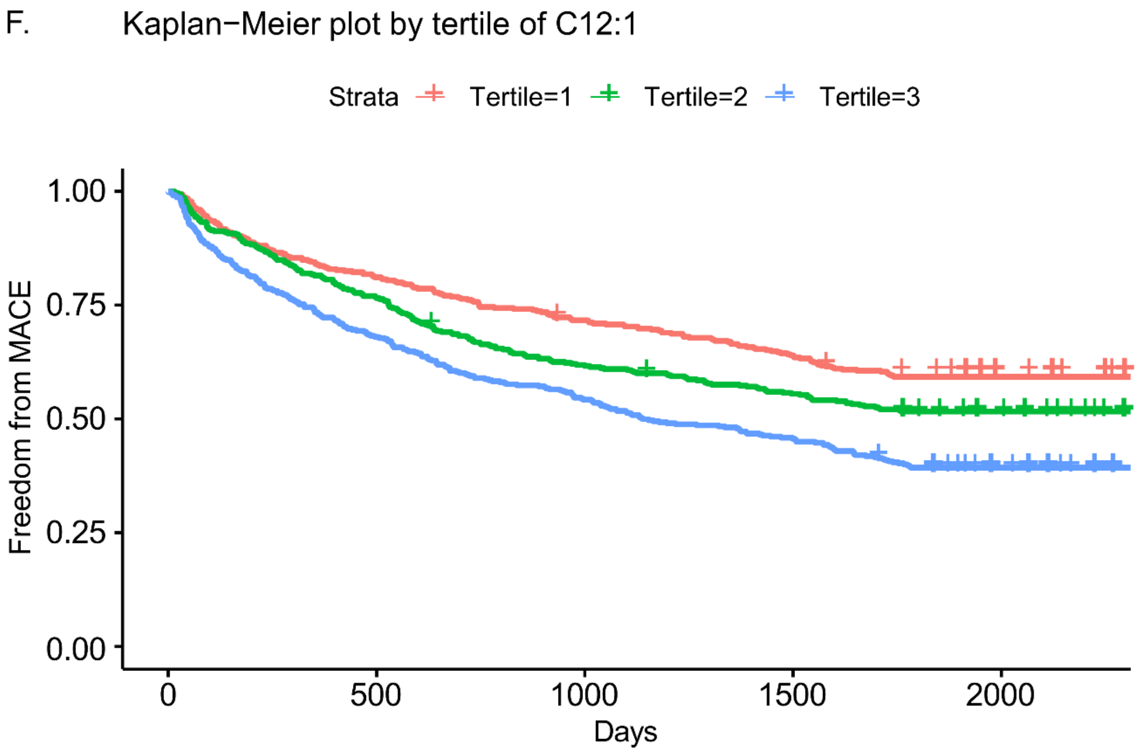
Kaplan−Meier plot by tertile of C12:1
Strata
Tertile=1
Tertile=2
Tertile=3
1.00
0.75
0.50
0.25
0.00
Freedom from MACE
0
500
1000
1500
2000
Days

G. Kaplan−Meier plot by tertile of C8

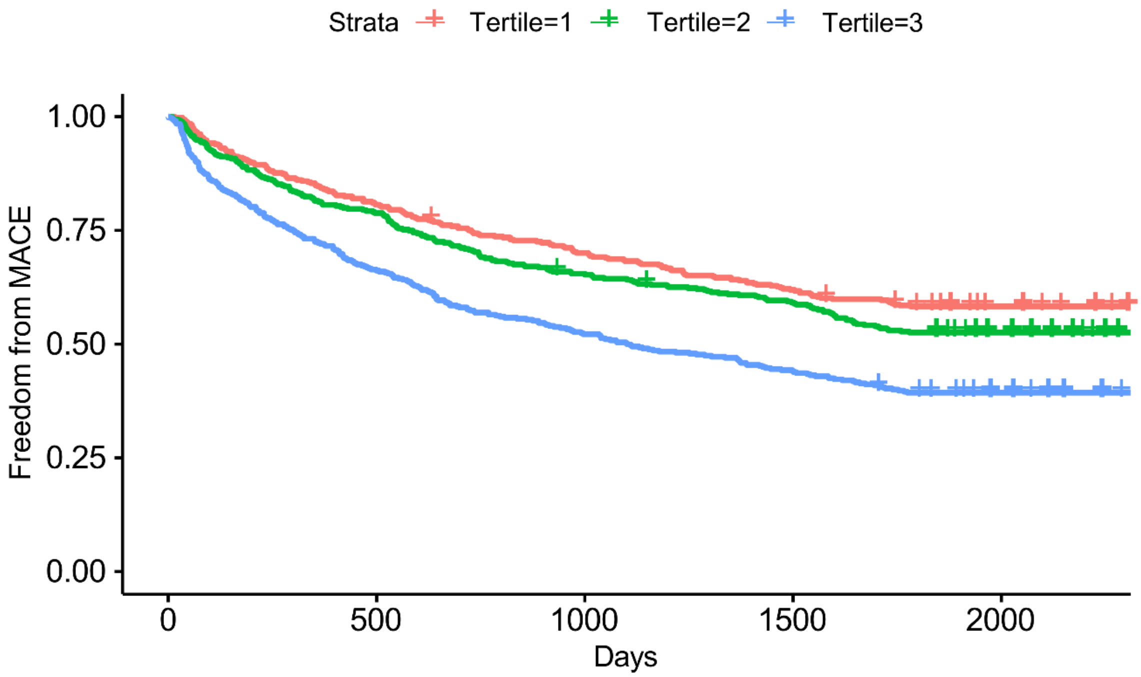

H. Kaplan−Meier plot by tertile of C16:2

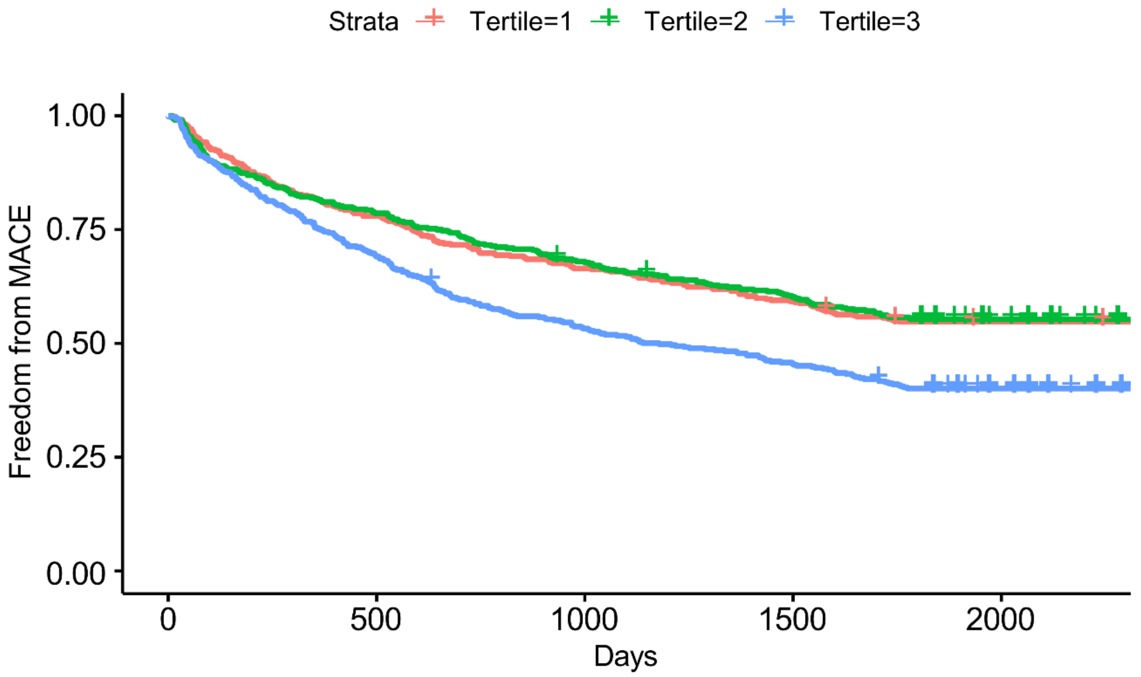

I.

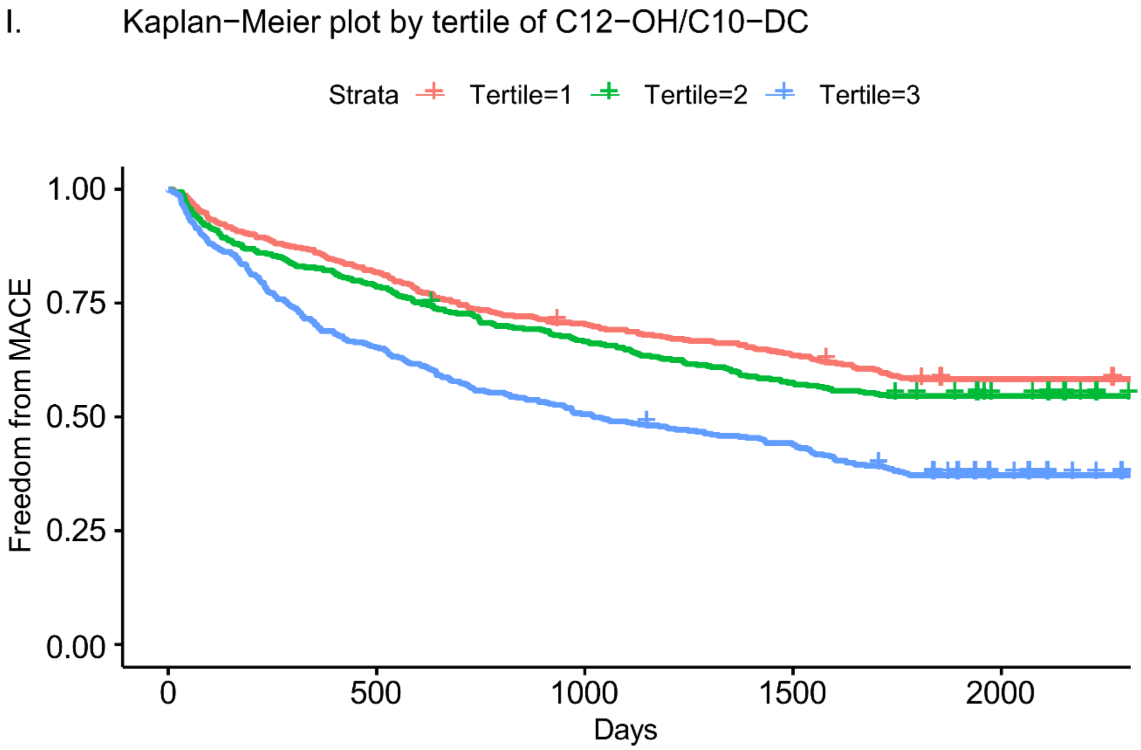

Kaplan−Meier plot by tertile of C12−OH/C10−DC
Strata
Tertile=1
Tertile=2
Tertile=3
Freedom from MACE
1.00
0.75
0.50
0.25
0.00
0
500
1000
1500
2000
Days

Supplemental Figure 2. Differences in absolute concentrations of nine acylcarnitines between MACE cases and non-MACE controls in TECOS, EXSCEL and CATHGEN cohorts.

Interquartile range plots of log-transformed absolute concentration levels of baseline metabolites for the nine metabolites significantly associated with MACE, stratified by MACE outcome in each cohort (TECOS N=996; EXSCEL N=978; CATHGEN N=1330).

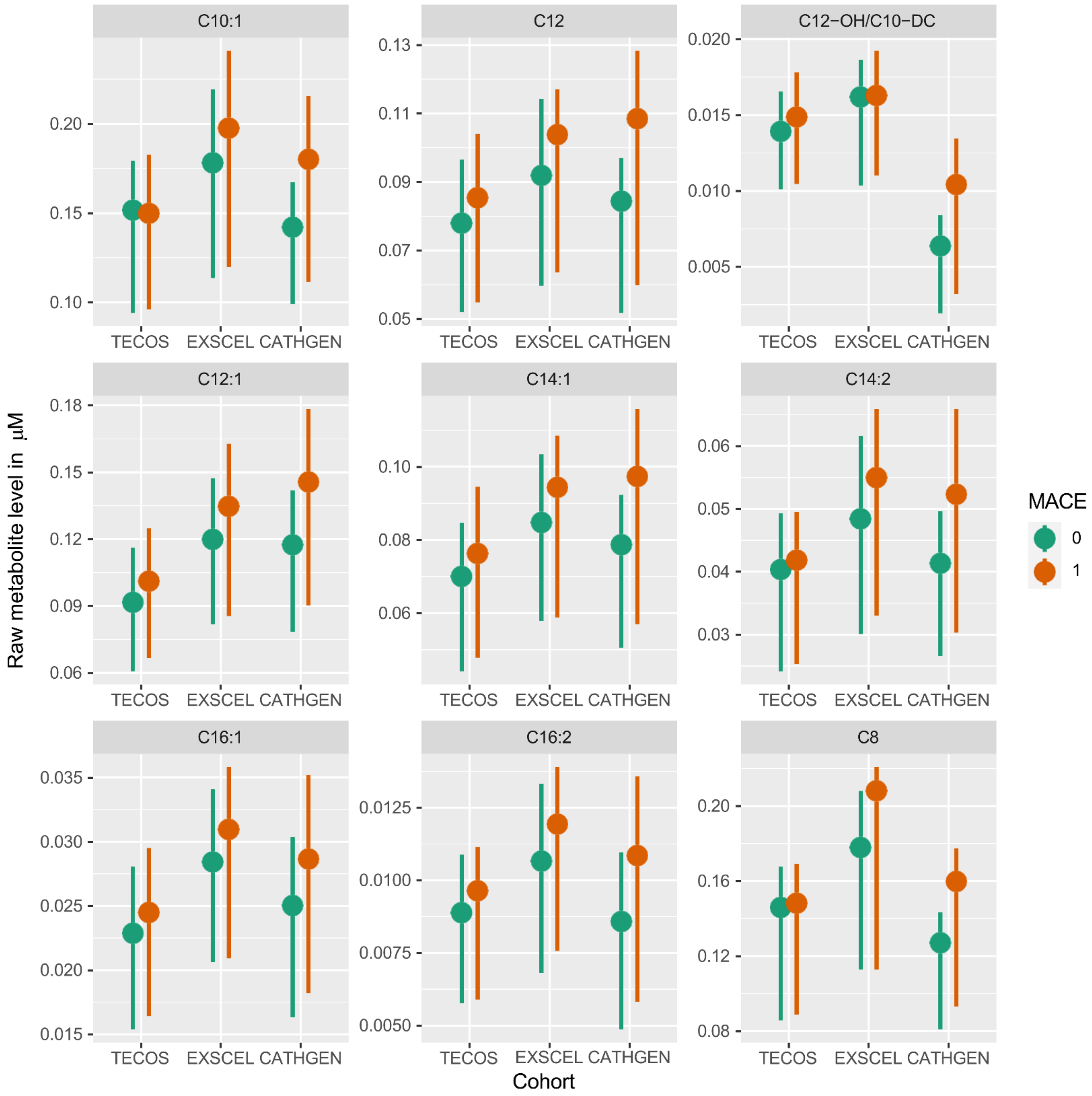

Supplemental Figure 3. Differences in the nine-metabolite composite score between MACE cases and non-MACE controls in TECOS, EXSCEL, and CATHGEN cohorts

Interquartile range plots of baseline log-transformed levels of the nine-metabolite composite score containing C8, C10:1, C12-OH/C10/DC, C12:1, C12, C14:1, C14:2, C16:2, C16:1 in MACE cases and non-MACE controls in each cohort (TECOS N=996; EXSCEL N=978; CATHGEN N=1330).

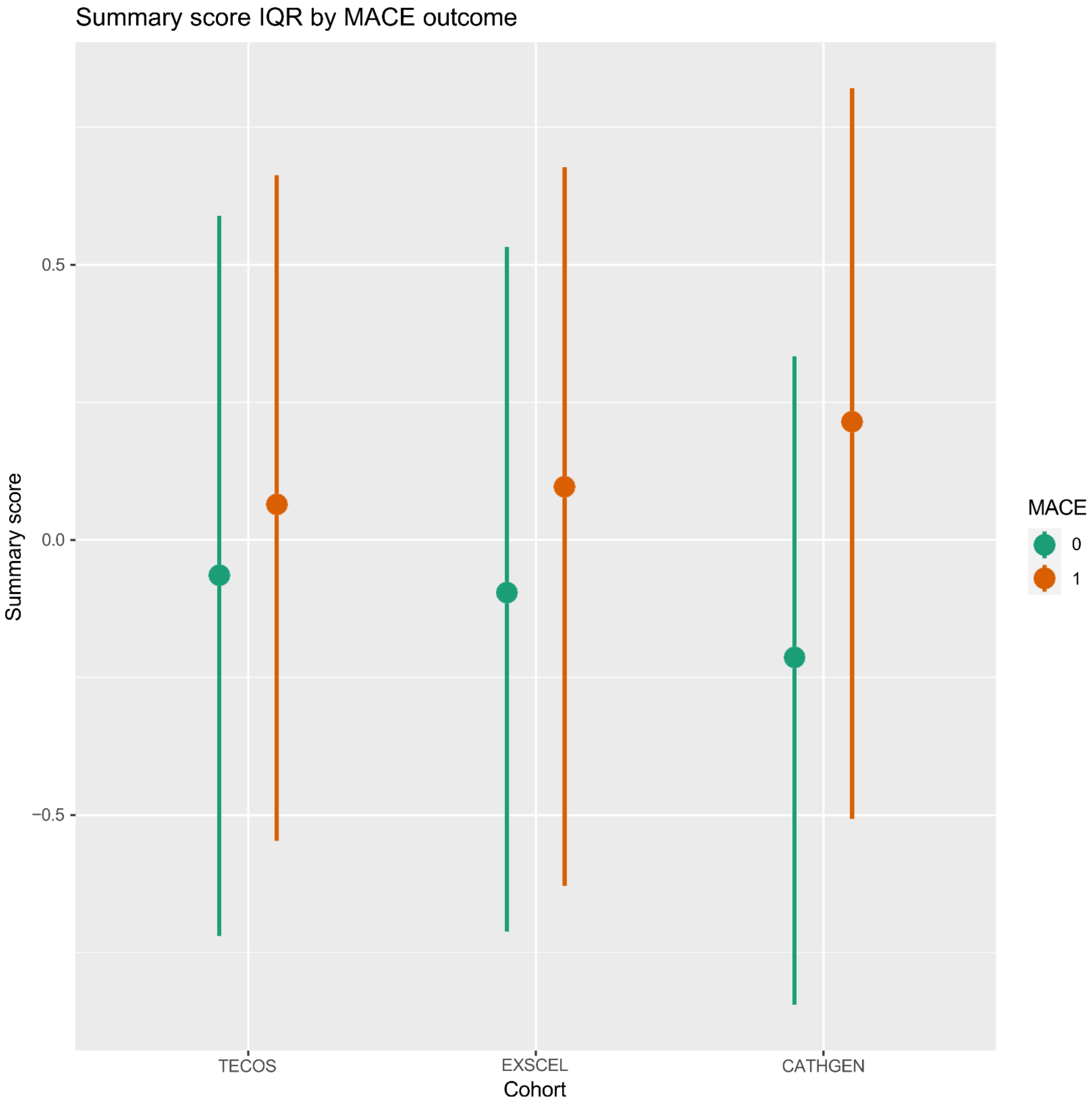

Supplemental Figure 4. Multivariate models for nine-metabolite score associated with MACE in meta-analysis.

Forest plot shows multivariate meta-analysis of the nine-metabolite score in TECOS (N=996) and EXSCEL (N=978). Nine metabolites included: C8, C10:1, C12-OH/C10/DC, C12:1, C12, C14:1, C14:2, C16:2, C16:1.

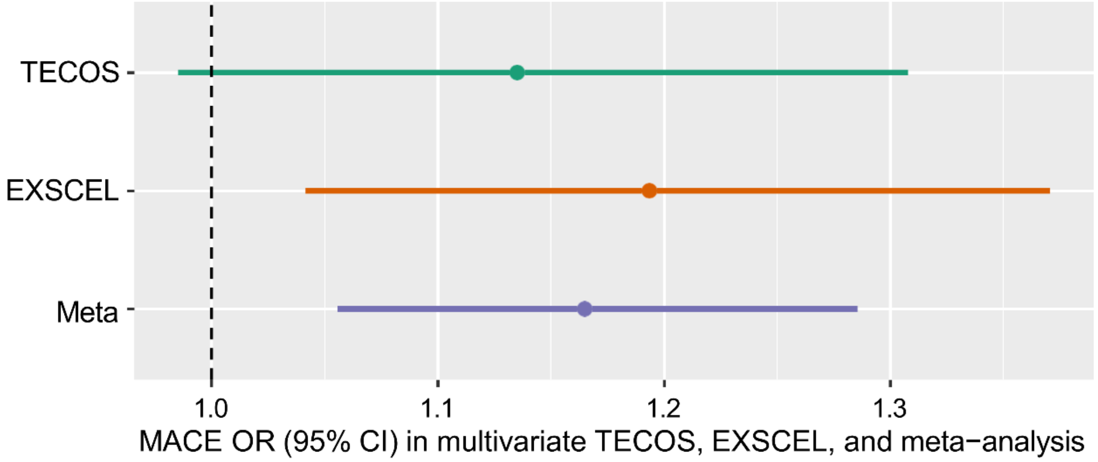

Supplemental Figure 5. Mitochondrial metabolites minimally improve discriminative capabilities for MACE prediction when added to the clinical model in CATHGEN

Receiver Operator Curve (ROC), Area Under Curve (AUC). Nine metabolites included in model: C8, C10:1, C12-OH/C10/DC, C12:1, C12, C14:1, C14:2, C16:2, C16:1. Clinical model includes age, sex, race, history of heart failure (HF), coronary artery disease (CAD), body mass index (BMI), hemoglobin A1C (HbA_{1c}), systolic blood pressure, creatinine, low density lipoprotein cholesterol (LDL-C) and smoking status (N=1330).

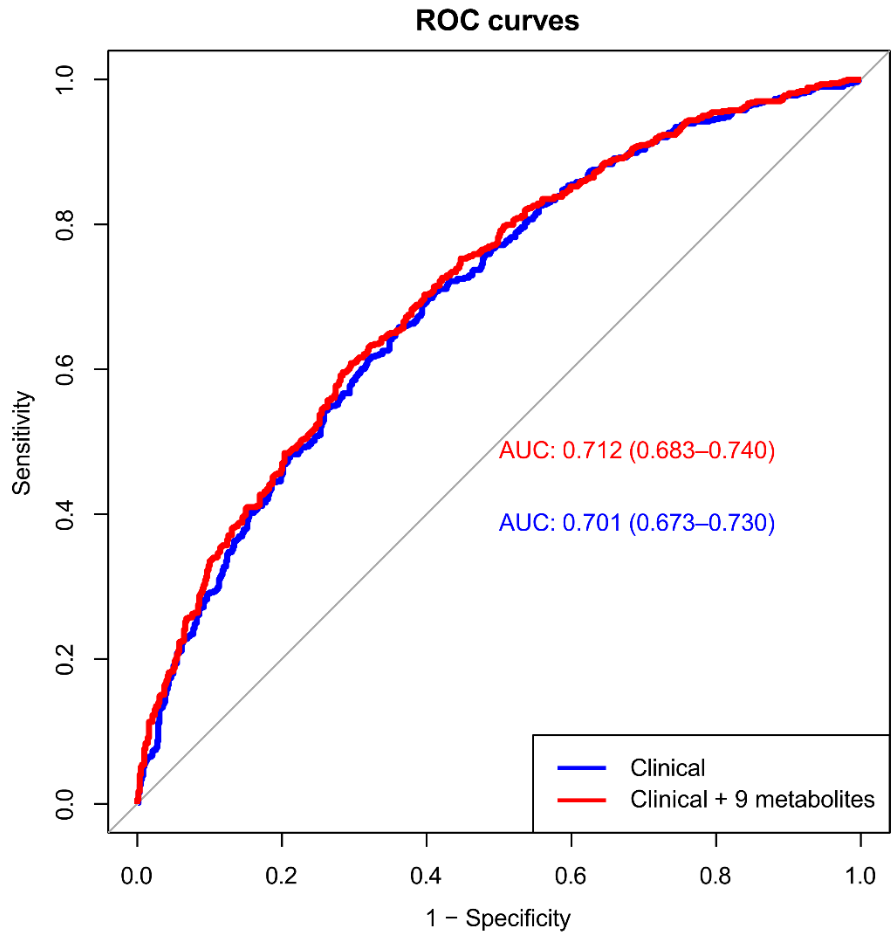

Supplemental Figure 6. Levels of 7 significant metabolites and of the summary score in exenatide and placebo arms of the EXSCEL studies, for baseline and 12-months

Box and whisker plots for individual metabolites with significant differences in exenatide treated participants compared to placebo randomized participants at 12 months and for the nine-metabolite summary score (N=973), p<0.05. EQW, exenatide every week.

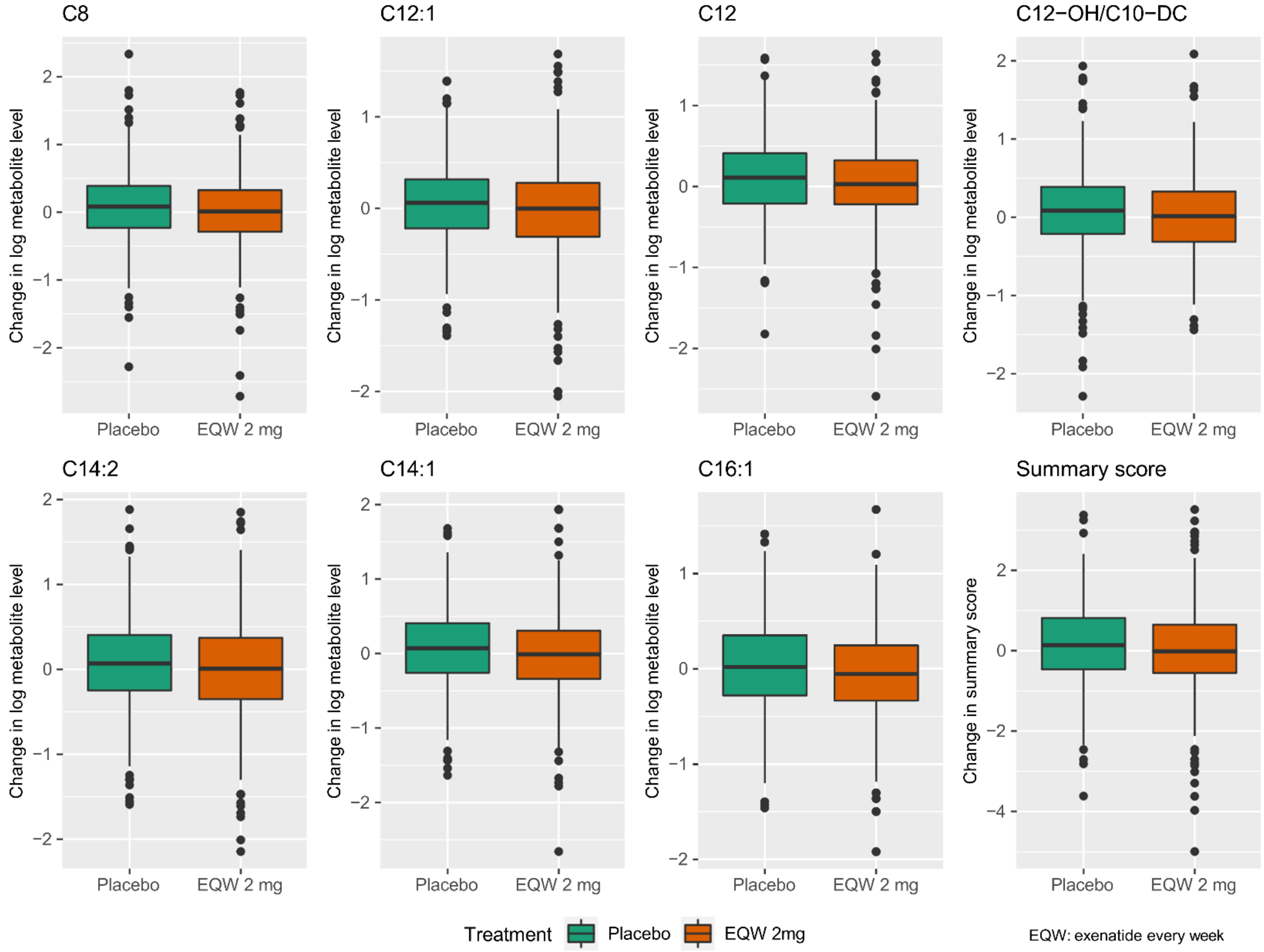

Supplement: Supplemental data [file jciinsight-8-168563-s139.pdf]
